# Tracking and changing beliefs during social interaction: Where computational psychiatry meets cognitive behavioral therapy

**DOI:** 10.3389/fpsyg.2022.1010012

**Published:** 2022-10-07

**Authors:** Jennifer Pott, Leonhard Schilbach

**Affiliations:** ^1^Institut für Klinische Verhaltenstherapie, LVR-Klinikum Düsseldorf, Düsseldorf, Germany; ^2^Abteilung für Allgemeine Psychiatrie 2, LVR-Klinikum Düsseldorf, Düsseldorf, Germany; ^3^Medizinische Fakultät, Ludwig-Maximilians-Universität München, Munich, Germany

**Keywords:** beliefs, computational psychiatry, cognitive behavioral therapies, psychosis, mechanisms

## Introduction

Models of dysfunctional beliefs and belief systems have a long tradition in psychiatry and psychotherapy and have been used to explain and help treat various psychiatric disorders. In this article, we focus on the role of beliefs and belief updating in psychotic disorders, but also discuss how these phenomena extend into the normal and various other patient populations. In addition, we review insights from the field of “computational psychiatry,” an area of research that uses mathematical models to describe and mechanistically explain how beliefs are formed, maintained or changed over time. We close by describing how cognitive behavioral therapy (CBT) uses the notion of beliefs to help treat psychiatric disorders and how an integration with “computational psychiatry” and digital phenotyping may help to provide new perspectives.

## How beliefs can help to explain psychiatric disorders

Having beliefs about oneself and states of the world is indispensable for human life, because they allow us to constrain behavior even when we are faced with incomplete sensory information about the environment (Seitz, 2022). Here, beliefs can be defined as relatively stable accounts of what a subject holds to be true and anticipates to happen in the future, even though this typically takes the form of a probabilistic representation, because we can be more or less sure about something. These probabilistic representations are typically formed below awareness, but they powerfully influence emotions and actions in often predictable or sometimes even inflexible ways. Also, people tend to trust their beliefs and may even do so in the presence of conflicting evidence (Fletcher and Frith, 2009). In extreme cases, persons may even hold “fixed beliefs that are not amenable to change in light of conflicting evidence.” Such beliefs according to the Diagnostic and Statistical Manual of Mental Disorders, Fifth Edition (DSM-V) are characteristic of what in psychiatric terms would be described as a delusion. Delusions, in turn, are a common feature of schizophrenia and other so-called psychotic disorders that can cause a person to lose touch with reality.

Research has investigated whether these so-called positive symptoms in schizophrenia are related to abnormal perception and/or abnormal beliefs. Both appear to be relevant, but according to a seminal review by Fletcher and Frith (2009) can be traced back to the same underlying core abnormality, i.e, a disturbance in error-dependent updating of inferences and beliefs about the world, which can be conceptualized as a disturbed hierarchical Bayesian framework. In such a framework–as introduced above–a belief is the subjective probability that some proposition about the world is true. This probability is continually updated in light of new incoming sensory evidence. Abnormal belief formation occurs when beliefs are not updated appropriately on the basis of new evidence (Hemsley and Garety, 1986). In line with these ideas, it has repeatedly been shown that persons with a diagnosis of schizophrenia show the tendency to jump to conclusions and to developed fixed beliefs more easily even in remission and when tasks are presented unrelated to delusional themes (e.g., Moritz and Woodward, 2005; Moritz et al., 2007). In the presence of psychotic-like experiences, persons seek less advice when making decisions (Scheunemann et al., 2021) and persons with a diagnosis of a psychotic disorder thought that a multitude of different interpretations of a given situation was plausible even when provided scarce or implausible explanations (Moritz and Woodward, 2004). Participants with psychotic disorder have also demonstrated both higher levels of certainty and a higher error rate in two studies on source attribution and the degree of subjective certainty for this judgement (Moritz and Woodward, 2002; Moritz et al., 2003). Furthermore, a bias against disconfirmatory evidence has been demonstrated repeatedly as an additional potential mechanism for the development and persistence of delusional ideation (Moritz and Woodward, 2006; Woodward et al., 2006a,b, 2008; Veckenstedt et al., 2011).

## Beliefs seen through the lens of computational psychiatry

The new burgeoning field of “computational psychiatry” seeks to complement traditional, symptom-based diagnostic schemes in psychiatry with mathematical modeling in order to infer on the mechanisms which generate observed behavior and brain activity in psychiatric patients (Stephan and Mathys, 2014). Next to this theory- and mechanism-driven interpretation of computational psychiatry, another important trend has been to use so-called big data approaches and interrogate them in a pure data-driven manner by using machine learning algorithms (Huys et al., 2016). To address the topic of belief formation and updating in psychiatric conditions the theory-driven approach appears to be particularly well-suited, because it involves using mathematical models that formally describe the cognitive processes, including beliefs and their probabilities, that underlie observable behavior. To this end, a wide variety of models can be used, but two have found particularly widespread application: models of reinforcement learning and Bayesian inference. The latter approach has even give rise to the notion that the human brain can be described as a “Bayesian brain” that constructs and continuously updates a generative model of its sensory inputs (Knill and Pouget, 2004; Friston, 2010).

**Figure 1:**
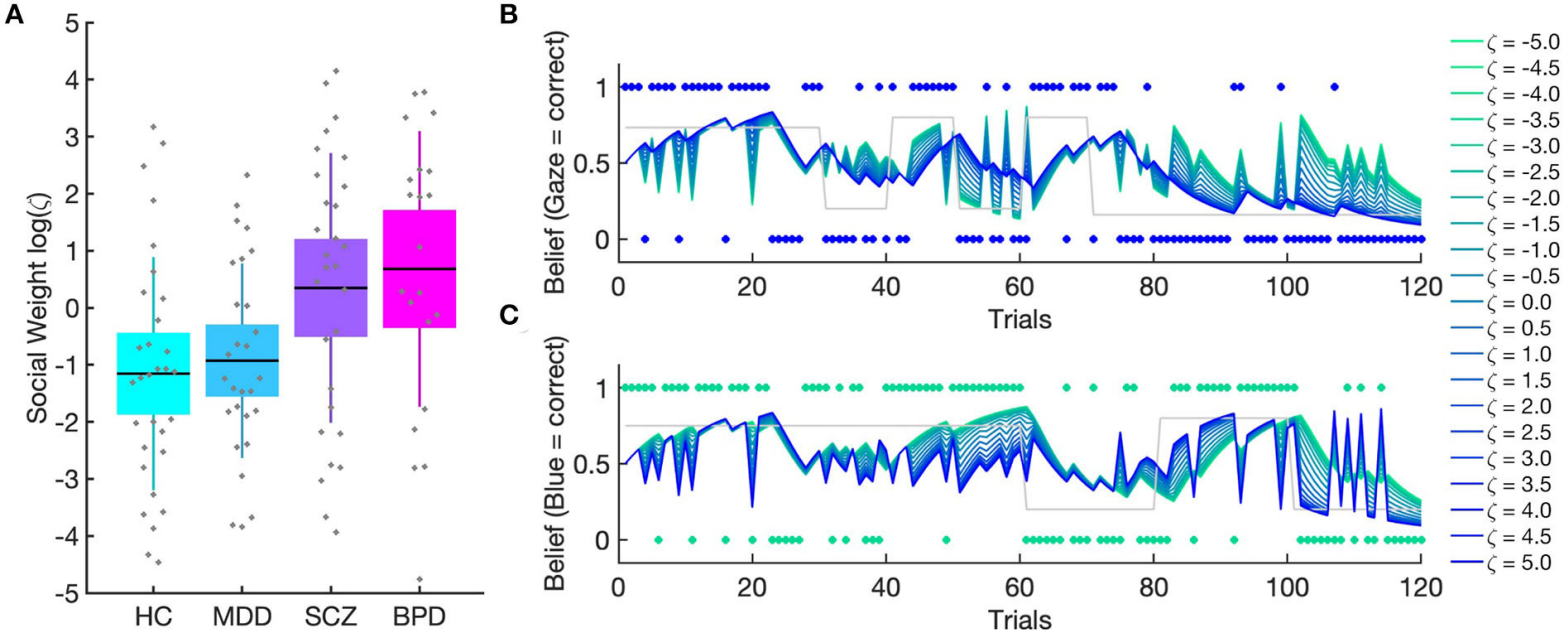
(Taken from: Henco et al., 2020). **(A)** Patients with borderline personality disorder (BPD) and schizophrenia (SCZ) gave social information more weight compared to healthy controls (HC) and patients with major depressive disorder (MDD). Boxes mark 95% confidence intervals and vertical lines standard deviations. **(B)** Simulations demonstrate the impact of varying weighting factor on combined belief. **(B)** shows that the combined belief of agents with high zeta values is aligned with the social input structure (blue dots) whereas these agents show a stochastic belief structure with regard to the non-social input structure (green dots) in **(C)**. Conversely, agents with low zeta values show a belief structure more closely aligned with the non-social input structure and a stochastic belief structure with regard to the social input as shown in **(B)**.

According to this approach, behavioral and neuroimaging studies are conducted that, for instance, use probabilistic learning tasks that ask study participants to learn from different types of information. In one such study conducted in our lab, we used a task that required learning about the winning probabilities of two cards and about the probability of a face giving the player advice by shifting gaze toward one of the two cards (Henco et al., 2020). Importantly, we did not explicitly tell participants to learn about the social information. The two types of information (non-social and social) were varied independently of each other during the course of the experiment, thereby constituting a volatile context, in which study participants with three major and severe psychiatric disorders were investigated: major depression (MDD; *n* = 29), schizophrenia (SCZ; *n* = 31) and borderline personality disorder (BPD; *n* = 31). In addition a group of participants was investigated without a history of a psychiatric disorder (*n* = 34). In other words, the study investigated whether volatility and probability learning is equally affected when inferring on the hidden states of non-social and social outcomes across the three different patient groups. We used the so-called hierarchical Gaussian filter (HGF; Mathys et al., 2011) to obtain a profile of each participant's particular way of updating beliefs when receiving social and non-social information while making decisions and selecting one of the cards on each trial. The HGF is a generic hierarchical Bayesian inference model for volatile environments with parameters that reflect individual variations in cognitive style. We went beyond other recent computational psychiatry studies using the HGF by using two parallel HGF hierarchies for social and non-social aspects of the environment. Our modeling framework was, thus, specifically designed to quantify the relative weight participants afforded their beliefs about the predictive value of social compared to non-social information. We found that patients with SCZ and BPD showed significantly poorer overall performance compared to healthy participants and patients with MDD, which raises the question which mechanisms underlie these patterns of behavior. Here, mathematical modeling allowed insights into how beliefs are updated and how these beliefs are translated into decisions: Results demonstrated revealed that SCZ and BPD patients both weighted their social-domain predictions more strongly than healthy study participants and patients MDD (Figure 1). This explains the lower performance of BPD and SCZ patients. Their stronger reliance on social cues was detrimental because the social cue was more volatile than the non-social one. The commonality of over-weighting social-domain predictions in SCZ and BPD patients suggests itself as the decision-making aspect of a general interpersonal hypersensitivity in both conditions. This is also reflected in excessive, albeit inaccurate, mental state attributions (also described as hypermentalizing) that are often observed in patients with BPD and SCZ.

Hypermentalizing is also a possible explanation for the findings by Seow and Gillan (2020), who used similar modeling to show that healthy participants at the high end of the paranoia spectrum used similar weighting of social information irrespective of whether incorrect advice was framed to be intentional or not, while low-paranoia participants reduced their social weighting when negative advice was cued to be intentional. This indicates that dysfunctional belief systems also play a role in the normal population and may present a dimensional continuum, on which different individuals can be placed. Interestingly, environmental changes also seem to affect belief systems and belief updating: Research by Suthaharan et al. (2021) has demonstrated that the initial phase of the COVID pandemic in 2020 increased individuals' paranoia and made their belief updating more erratic. This was examined by combining self-rated paranoia scores and computerized social and non-social belief updating tasks. Here, it was found that the increase in self-rated paranoia was less pronounced in US states that enforced a more proactive lockdown and more pronounced at reopening in states that mandated mask-wearing. Computational modeling revealed that certain types of behavior (win-switch) and volatility priors tracked these changes in self-reported paranoia with policy.

Taken together, computational psychiatry provides important new tools to investigate the mechanisms that underlie cognition and behavior. By doing so, computational psychiatry aims at establishing a new and mathematically formalized way of assessing beliefs, how they change over time and how they relate to subjective experience and observable behavior. This powerful approach also holds great potential for the investigation of the neurophysiology related to psychiatric conditions and may inform differential diagnosis and subgroup detection in accordance with what has been described as personalized or precision psychiatry (Stephan and Mathys, 2014; Friston et al., 2017). In addition, it has more recently been suggested that computational psychiatry could also play an important role in elucidating the computational mechanisms of cognitive and behavioral psychotherapeutic interventions, which often aim at changing a person's beliefs in order to alleviate symptoms and treat psychiatric conditions (Moutoussis et al., 2018; Nair et al., 2020; Smith et al., 2021).

## Discussion and outlook: How beliefs (and changing them) can help to treat psychiatric disorders

The cognitive turn of what at the time was still described as “behavioral therapy” consisted in introducing the idea that beliefs play an important role in mediating between so-called “activating events” and “emotional consequences” (Ellis, 1958). In addition, the notion of “cognitive distortions” was introduced to indicate that beliefs can be unhelpful of even distorted (Beck, 1963). Such unhelpful beliefs can lead to negative emotions and maladaptive actions, thereby forming what has become known as a cognitive triangle (belief–affect–behavior). Reviewing the complete history of what is today referred to as “cognitive behavioral therapy” (CBT) and studies to investigate its effects and underlying mechanisms clearly is beyond the scope of this article. But it is safe to say that a large and increasing body of literature indicates that CBT techniques such as disputation of beliefs and cognitive restructuring are efficient and allow to target the general and specific belief systems that are deemed relevant for different psychiatric conditions. With regard to psychotic disorders, in particular, it has been demonstrated that meta-cognitive training (MCT) is a novel cognitive approach geared toward the treatment of positive symptoms in psychosis, but also other clinical conditions. MCT tries to help individuals experiencing psychosis to become more aware of the beliefs involved in their illness and to counteract the biased beliefs and assumptions that may predispose an individual to develop delusions (see Moritz et al., 2022 for a recent overview). Importantly, MCT typically takes place in a group setting, which allows for social interaction and exchange between different persons with the aim to reflect upon experiences and thoughts from different perspectives and consider information provided by others. In fact, the social interactions between group members are seen as a crucial aspect and key to the learning process.

Consistent with this, it is well-known that the social interaction between patient and therapist and the so-called therapeutic relationship plays a fundamental role in contributing to the success of psychotherapy (Leahy, 2008). In this regard, the concept of a so-called “complementary therapeutic relationship” has been proposed (Caspar et al., 2005), which suggests that therapists are “supposed to offer each patient an individually custom tailored relationship that suits his or her important goals.” In other words, the therapist should adjust to the interactional profile and needs of the patient in order to contribute to and allow for a smooth and harmonious interaction, which lays the foundation for a helpful therapeutic relationship by promoting the development of trust. This also resonates with findings from non-clinical populations, where it has been demonstrated that the degree of interpersonal similarity is closely related to relationship quality (Bolis et al., 2021). In other words, how well/little people match interpersonally is important for the success of social interaction and communication, both in a non-clinical and a clinical context. This has been described as the “social interaction mismatch hypothesis” and can help to guide studies in social neuroscience toward the investigation of cross-brain processes (see Redcay and Schilbach, 2019 for a review). In a therapeutic context it is often a requirement that the therapist adjusts to the interactional needs of the patient to create a therapeutic relationship that later on can also be used to initiate change and to help the patient make so-called corrective experiences that challenge one's fears or expectations.

In addition, it can be argued that “disorders of social interaction” represent a defining feature of psychiatric disorders (Schilbach, 2016) and that addressing social interaction difficulties as a transdiagnostic phenomenon constitutes an important therapeutic goal (Schilbach et al., 2022).

With regard to the dyadic micro-processes relevant for the establishment of a helpful, motivating and trusting therapeutic relationship, it has been demonstrated that engaging in joint attention and sharing experiences with another person–even outside a therapeutic context – recruits reward-related neuro-circuitry, which can be interpreted in terms of an intrinsic motivation for social connection (Schilbach et al., 2010; Pfeiffer et al., 2014). Moreover, results from a wide range of studies demonstrate that non-verbal synchrony in dyadic interactions plays an important role to create rapport and may act as a “social glue” that binds persons together (Schilbach et al., 2008; Neufeld et al., 2016; for a review see Schilbach, 2015). Consistent with these ideas, results from a study by Ramseyer and Tschacher (2011) have shown that differences in synchrony at the level of nonverbal behavior are linked to relationship quality and therapy outcome (see also Koole and Tschacher, 2016 for a review). In this regard, the advent of novel technologies and methodologies now allows for a more fine-grained and quantitative analysis of interpersonal behavior during dyadic social interactions (Lahnakoski et al., 2020). Here, it has been found that it is not only synchrony that matters, but that aspects of interpersonal orienting and distance also predict the subjective quality of social interactions. Combining these new methods with computational modeling and other approaches from computational psychiatry promises to provide completely new insights into the mechanisms of social interaction and into how beliefs are (sometimes) shared across different brains (Henco and Schilbach, 2021). Furthermore, it appears feasible to investigate how differences in nonverbal synchrony during a social interaction may influence beliefs a person holds. Here, the dynamics of a social interaction could help to consolidate or change previously acquired social beliefs by providing a form of social feedback and validation. In combination with experimental tasks that record psychophysiology from two interacting persons, these developments could help to increase our understanding of how to improve the relationship quality and efficacy of psychotherapeutic interventions in the future (Bolis et al., 2017).

In summary, beliefs play a prominent role in our daily lives and help us to successfully navigate the environment and social interactions by providing probabilistic estimates of what we can hold to be true. But beliefs can also lead us astray and cause intense suffering as evident in psychotic disorders, but also a wide range of other psychiatric conditions. Fortunately, beliefs–in many, if not all instances–are subject to change or can be recognized as just that, beliefs. Consequently, we may even show adaptive behavior in the presence of unhelpful beliefs and can make new experiences - often during social interactions - that may help us to leave certain beliefs behind.

## Author contributions

All authors listed have made a substantial, direct, and intellectual contribution to the work and approved it for publication.

## Funding

This paper is funded by Rüdiger Seitz, via the Volkswagen Foundation, Siemens Healthineers, and the Betz Foundation.

## Conflict of interest

The authors declare that the research was conducted in the absence of any commercial or financial relationships that could be construed as a potential conflict of interest.

## Publisher's note

All claims expressed in this article are solely those of the authors and do not necessarily represent those of their affiliated organizations, or those of the publisher, the editors and the reviewers. Any product that may be evaluated in this article, or claim that may be made by its manufacturer, is not guaranteed or endorsed by the publisher.
